# Development and Applications of Viral Vectored Vaccines to Combat Zoonotic and Emerging Public Health Threats

**DOI:** 10.3390/vaccines8040680

**Published:** 2020-11-13

**Authors:** Sophia M. Vrba, Natalie M. Kirk, Morgan E. Brisse, Yuying Liang, Hinh Ly

**Affiliations:** 1 Department of Veterinary & Biomedical Sciences, University of Minnesota, Twin Cities, St. Paul, MN 55108, USA; vrbax006@umn.edu (S.M.V.); liangy@umn.edu (Y.L.); 2 Comparative Molecular Biosciences Graduate Program, Department of Veterinary & Biomedical Sciences, University of Minnesota, Twin Cities, St. Paul, MN 55108, USA; kirk0332@umn.edu; 3 Biochemistry, Molecular Biology and Biophysics Graduate Program, Department of Veterinary & Biomedical Sciences, University of Minnesota, Twin Cities, St. Paul, MN 55108, USA; briss049@umn.edu

**Keywords:** viral vectored vaccines, veterinary vaccines, COVID-19, HIV-1, influenza, HPV, Ebola, wildlife, zoonotic disease, disease control

## Abstract

Vaccination is arguably the most cost-effective preventative measure against infectious diseases. While vaccines have been successfully developed against certain viruses (e.g., yellow fever virus, polio virus, and human papilloma virus HPV), those against a number of other important public health threats, such as HIV-1, hepatitis C, and respiratory syncytial virus (RSV), have so far had very limited success. The global pandemic of COVID-19, caused by the SARS-CoV-2 virus, highlights the urgency of vaccine development against this and other constant threats of zoonotic infection. While some traditional methods of producing vaccines have proven to be successful, new concepts have emerged in recent years to produce more cost-effective and less time-consuming vaccines that rely on viral vectors to deliver the desired immunogens. This review discusses the advantages and disadvantages of different viral vaccine vectors and their general strategies and applications in both human and veterinary medicines. A careful review of these issues is necessary as they can provide important insights into how some of these viral vaccine vectors can induce robust and long-lasting immune responses in order to provide protective efficacy against a variety of infectious disease threats to humans and animals, including those with zoonotic potential to cause global pandemics.

## 1. Introduction

Current Food and Drug Administration (FDA)-approved vaccines include live attenuated, inactivated, and subunit vaccines for a variety of human diseases [1]. In general, a live attenuated vaccine is made by attenuating the virulent nature of a pathogen while keeping a certain level of its replication competency. Current examples of live attenuated vaccines include smallpox, MMR combined vaccine (measles, mumps, rubella), chickenpox, and yellow fever. Inactivated vaccines are replication-deficient or killed viruses or bacteria that are administered in order to create immunological memory to a particular vaccine antigen (immunogen), or even a toxin, from the pathogen. Current examples of inactivated vaccines include hepatitis A, annual (seasonable) influenza vaccine, polio vaccine, and rabies vaccine. Another commonly used vaccine design approach is subunit vaccines, which contain certain antigens from the pathogen that can stimulate a protective immune response against the pathogen when administered as a vaccine. For a complete list of FDA-approved vaccines, please follow this web-link: https://www.fda.gov/vaccines-blood-biologics/vaccines/vaccines-licensed-use-united-states.

Despite recent advances in vaccinology, there are still many infectious diseases such as AIDS, malaria, and hepatitis C that still do not have FDA-approved vaccines despite decades of intense investigation. The lack of a vaccine for these and other intractable diseases can be explained by many different factors that include but are not necessarily limited to the unique properties of the individual pathogen, lack of relevant animal models or economic incentives to produce and test a vaccine, and/or insurmountable regulatory and/or ethical issues associated with vaccine-associated clinical trials, especially in pediatric and immunocompromised human populations [2]. However, another more simplistic reason may include the inability of traditional vaccine design methods to produce a functionally protective vaccine. Therefore, it is important to consider new technologies for vaccine design and development.

A technology that has shown great promise is the use of viral vectors as vehicles to deliver the desired immunogens. Viral vectors were first developed for vaccine applications almost forty years ago when vaccinia virus (VACV) was used as a vector to express the hepatitis B surface antigen HBsAg [3]. When this vector was tested in chimpanzees for protective immunity, it was shown to be sufficient to protect the animals against hepatitis B infection [4]. Since this promising first application of viral vectors for vaccine development, a variety of new viral vectors have been created using prototypic viruses from many different virus families (Table 1). These virus vectors were optimized to improve their genomic packaging capacity, cellular tropisms, and replication capabilities in order to customize the desired immune responses. Some of these viral vectors have been used for vaccine applications not only in human medicine but also in veterinary settings [5,6]. The levels of success of virus-vectored vaccines in veterinary medicine, however, have not been replicated in human clinical settings as only one viral vector-based Ebola vaccine (ERVEBO^®^) has been FDA-approved for human use (Table 2 and Table 3). These and other issues described in this section will be discussed in some depth below. Presently, the challenge of translational applications of viral vectored vaccines in humans is largely due to safety concerns of potential recombination (or reversion) events of the genomes of the virus vectors with those of circulating viruses to generate a virulent phenotype as well as the technical challenges of scaling up the process of virus vectored vaccine production. Despite these formidable challenges, several unique features of viral vectors, including their ability to induce long-lasting immune responses, continue to drive their development and application. This review covers a wide variety of promising viral vaccine vectors, their recent developments and general designs, with a particular focus on viral vectors that have not been extensively reviewed elsewhere previously.

## 2. General Concepts and Approaches to the Development of Viral Vectors

There are four common approaches to the development of viral vaccine vectors, which include methods to produce replication-competent (RC) viral vectors, replication-defective (RD) viral vectors, single-cycle (SC) viral vectors, and multi-segmented (MS) viral vectors. The general strategies for their development will be discussed in this section, with more specific examples of the different viral vectored vaccines being detailed in separate sections thereafter.

An attractive aspect of viral vectors for use in vaccine development is their ability to replicate (reproduce) in the proper host cells. An RC viral vector is one that is capable of infecting cells, replicating (duplicating) its genetic information, and creating new viral progeny that can then infect new cells. A major advantage with this type of viral vector is its ability to amplify the vaccine antigen (immunogen) that is built into the genome of the viral vector. By replicating, the viral vector is able to amplify not only its genome but also the immunogenic gene of interest. Studies performed on four serotypes of RC adenovirus (RC-Ad) demonstrated that each of these viral vectors could produce 10^3^ to 10^5^ infectious particles per cell [10,36]. This creates a large amount of the vaccine immunogen to drive an effective adaptive immune response [10]. However, there are drawbacks associated with these RC-Ad vectors due to their potential to cause unintended side effects, especially in immunosuppressed or immunocompromised individuals [37]. In order to alleviate some of these concerns, RC viral vectors, including RC-Ad [38], have been attenuated through deletion of certain viral genes, such as the viral intrinsic immunomodulating genes encoded in the wild-type virus genome [24,25,39]. Through these strategies, RC viral vectors have shown improved safety profiles, but the potential for these virus vectors to cause undesirable side effects still needs to be carefully monitored.

On the other hand, RD viral vectors have been used for a variety of vaccine applications due to their excellent safety profiles. Because these RD viral vectors are unable to replicate, they do not produce infectious viruses. Typically, to make the viral vector replication-defective, one or more genes required for viral genome replication, synthesis, and/or assembly are deleted. The virus is then propagated in the complementing cell line that expresses the missing viral gene product(s) in trans [40]. Even though their replication is restricted, RD viral vectors are still able to express the desired immunogen in order to induce innate and adaptive immune responses that are generally localized to the site of RD viral vector administration [40]. The heterologous (immunogenic) gene(s) expressed by the RD viral vectors are presented through the major histocompatibility complex MHC class I and class II pathways. This effectively stimulates the adaptive arm of the immune response. In addition, RD viral vectors are thought to activate innate immune sensor pathways, such as toll-like receptors, thereby acting as their own adjuvants [40]. The strengths and specifics of the immune response elicited by RD viral vectors depend on the vector used, which will be detailed in later sections. Upon comparing the vaccine potency of candidate viral vectors for the Ebola virus (EBOV), RD-Ad vaccines and RC vesicular stomatitis virus (RC-VSV) vaccines both elicited strong immune responses despite RD-Ad being unable to replicate, which supports the promise of these RD vectors [40]. After considering phase I trial data with a remarkable rate of protection (almost 100%) in ring vaccination trials [41], VSV-ZEBOV was selected to continue in development and became the only available viral vector-based vaccine on the market [42], which is now known as ERVEBO^®^ (rVSVΔG-ZEBOV-GP) vaccine, produced by Merck & Co. [43]. However, the success of RD viral vectors is not unanimous and some RD viral vectors, such as modified vaccinia virus Ankara (MVA), have been found to induce limited immune responses in clinical trials [44]. In summary, while RD viral vectors represent a safer alternative to RC viral vectors, further development is needed for certain RD viral vectors in order to ensure that they can induce sufficient protective immune responses.

Another viral vector platform consists of the single-cycle virus (SC) vectors that are able to replicate and amplify the inserted heterologous gene but do not express the viral late genes needed for making functional progeny virions due to their targeted deletion from the virus genome. Instead, virus particles are produced by transfecting cells that express these viral late genes in trans. When given as a vaccine, these virus particles can infect their target cells but are not able to produce virus particles and instead transcribe only viral RNA [10]. Therefore, this SC viral vector has a better biosafety profile as it does not continuously produce infectious virus with the potential to cause adverse side effects or diseases in certain vulnerable populations [45]. In studies of a green fluorescent protein (GFP)-encoding SC-Ad vector in macaques, SC-Ad6 induced high levels of anti-GFP antibodies and T cell responses [45]. Overall, SC viral vaccine vectors are generally good approaches for vaccine development as they have fewer safety risks than RC viral vectors and can amplify the heterologous gene (immunogen) through a single cycle of virus replication, which is lacking by the RD viral vectors.

A new generation of viral vaccine vectors includes those viruses with a multi-segmented (MS) RNA genome. The genome of these MS viral vectors contains multiple genomic strands that encode for more than one viral gene product and therefore can also accommodate multiple vaccine antigen(s). Along with expanding the repertoire of protein antigens (immunogens) that can elicit effective immune responses, a major advantage of the MS viral vaccine vectors is the attenuating features that allow for an increased safety profile. These unique characteristics have recently been demonstrated by the tri-segmented reverse genetics systems of arenaviruses, such as the lymphocytic choriomeningitis virus (LCMV) [21,22] and Pichinde virus (PICV) [23].

## 3. Expansive Repertoire and Selection of Currently Available Viral Vectors for Vaccine Development

### 3.1. Retrovirus and Lentivirus Vectors

Viral vectors based on retroviruses and lentiviruses (Table 1 above) have long been used in gene therapy trials due to their specific and large genome packaging capacity, extensive attenuation, and ability to integrate the genetic information of the viral genome into the host cell genome [46]. Retroviruses have an RNA genome, which is transcribed into DNA through the action of an enzyme called reverse transcriptase. In addition to reverse transcriptase, retroviruses encode the integrase enzyme, which mediates integration of the viral reverse-transcribed DNA genome into the host chromosome, and a protease enzyme that mediates viral protein processing in order to produce a mature and infectious virus [47]. Lentiviruses (e.g., HIV and human T-lymphotropic virus) belong to a genus of retroviruses that is characterized by long incubation periods and latency in the infected hosts, which can lead to chronic disease progression. Vectors based on retroviruses, such as the Moloney murine leukemia virus (MoMLV), were the first retroviral vectors to be developed, as well as some of the first used in gene therapy applications [48]. Retroviral and lentiviral vectors have mostly been used in applications where gene integration is favorable and desirable, such as in the case of gene therapy to correct a faulty (defective) host gene [49,50]. However, in order to make these retroviral vectors amenable as viral vaccine vectors, the viral envelope and several regulatory genes (in the case of lentiviruses) are deleted [50,51,52,53,54]. These viral vectors can be pseudotyped to express the envelope glycoprotein of another virus, such as the G protein of VSV, which can help to broaden the host cell range (tropism) of the viral vectors [7]. While there are some concerns for unintended insertional mutagenesis and malignant transformation mediated by lentiviral vectors, no reports have been presented about these phenomena to date [7]. Therefore, applications of retrovirus- and lentivirus-based vaccine vectors have been extensively used and reviewed elsewhere [7,8,9].

### 3.2. Adenovirus Vectors

Adenoviruses have a linear, double-stranded DNA genome and are non-enveloped with an icosahedral capsid. A number of viruses from the *Adenoviridae* family have been engineered for use as viral vectors, mainly because adenoviruses have a broad cellular tropism, are generally species-specific, and can accommodate and stably express large inserts of up to 8 kB of heterologous gene [26]. These viral vectors (Table 1) are based on both human and non-human adenoviral species in response to concerns of preexisting immunity to human adenoviruses when they are used as viral vaccine vectors [11,12]. As these viral vectors have been extensively reviewed elsewhere [10,11,12,13,14], we will only briefly discuss applications of the SC-Ad vectors here.

SC-Ad vectors mitigate the safety concerns of RC-Ad vectors as a result of the deletion or selective repression of several viral genes involved in capsid assembly in order to prevent production of an infectious virus [55] while still retaining significantly more immunogenicity than RD-Ad vectors in the vaccinated hosts [40]. An SC-Ad vector that has been extensively tested is the SC-Ad6, which is based on adenovirus serotype 6 (Ad6). Ad6 was utilized as it has a lower seroprevalence rate in the general human population when compared to other Ad serotypes, such as Ad5 [45]. To create this SC-Ad6 viral vector, the gene for the IIIa capsid cement protein, which aids in capsid assembly following viral genome replication, was deleted [37,45]. In a direct comparison between SC-Ad, RC-Ad, and RD-Ad through a luciferase assay at day 3 after immunization, the SC-Ad and RC-Ad vectors expressed luciferase 38 and 73 times higher than RD-Ad6, respectively, and as such, both SC-Ad and RC-Ad can be more effective than the RD-Ad to amplify heterologous genes to drive robust immune responses against the vaccine antigens [45]. This SC-Ad6 has been used in a variety of preclinical animal models [56,57] and consistently showed that SC-Ad6 could stimulate better immune responses than RD-Ad6 [45]. Specifically, SC-Ad6 vectors induced high levels of serum antibodies and provided protection long after the first immunization [56]. Specifically, in a study of the SC-Ad expressing EBOV glycoprotein (GP), intranasal administration of the vaccine to Syrian hamsters generated EBOV GP-specific antibodies that peaked at 17 weeks after a single vaccination. Antibody levels remained significantly elevated beyond 6 months in comparison to controls [56]. In preclinical testing of this vector, cotton rats immunized with SC-Ad6 expressing hemagglutinin (HA) of the influenza virus could significantly reduce influenza viral titers in lung homogenates 24 h after challenge [57]. Additionally, immunized Syrian hamsters displayed higher levels of hemagglutination inhibition (HAI) titers, which measure HA binding antibody levels, in sera and bronchoalveolar lavage (BAL) fluid seven weeks after administration of a single intranasal dose of SC-Ad [57]. These aspects argue for further development of the SC viral vectors, such as the SC-Ad, as they have the potential for implementation in future human vaccination trials.

### 3.3. Poxvirus Vectors

Viral vectors based on poxviruses were among the earliest eukaryotic viruses to be engineered to express heterologous genes and were the first to be used for vaccination purposes [15]. Today, the main poxvirus vector used for vaccination is the RD-MVA, which has been extensively reviewed elsewhere [15,16]. Other RD and RC vectors have been developed from the virus family *Poxiviridae,* including fowlpox virus and canarypox virus vectors (Table 1). These viruses have a linear, double-stranded DNA genome and are known for their ability to induce a strong adaptive immune response and strict regulatory control of heterologous gene expression [15,16]. Additionally, poxviruses can accommodate a much larger amount of heterologous DNA on their genomes (up to 30 kB) than many other viral vectors, making them particularly good candidates for use as viral vectors for the development of multi-valent and multi-pathogen vaccines [58].

Avipoxviruses, such as canarypox (ALVAC) and fowlpox virus (FWPV), show great promise as viral vectors. Similar to MVA, ALVAC and FWPV are able to accommodate insertions of a large amount of heterologous DNA (Table 1). The natural productive host range of avipoxvirus vectors are avian species, but studies on ALVAC and FWPV have shown that inoculation of mammalian cells with these vectors produces abortive infections [17,18]. A major advantage of ALVAC vectors is their ability to induce high immunogenicity in mammalian cells [59,60,61]. ALVAC vectors have been shown to induce distinctly strong proinflammatory immune responses and antiviral responses in stimulated human peripheral blood mononuclear cells (PBMCs) as compared to other poxvirus vectors [60]. Specifically, these ALVAC vectors stimulated macrophages and monocytes to secrete interleukin (IL)-1β and tumor necrosis factor (TNF)-α, T cells to secrete interferon (IFN)-γ, and plasmacytoid dendritic cells to secrete IFN-α2. As such, this ALVAC platform may be best applied to disease threats in which a Th1 immune response is desirable.

Due partly to this promising immunogenicity, ALVAC vectors have been used as a viral vector for vaccine development against Hendra virus [62] and HIV-1 [63] in preclinical and clinical studies, respectively. The HIV-1 clinical trial (RV144) showed that there were 31.2% fewer HIV-1 infections in the vaccinated group in comparison to the placebo group after three years of follow-up [64]. Although this efficacy level was not sufficient for licensing, it showed an exciting potential for HIV-1 vaccine development [63]. Upon further analysis of the vaccinated group, there were several main immune correlates associated with decreased risks of HIV-1 infection: IgG antibodies specific to the HIV-1 envelope (env) V1/V2 region (V1-V2 IgG3) and polyfunctional CD4+ T cell stimulation inducing CD40L, IFN-γ, tumor necrosis factor-α (TNF-α), IL-2, and IL-4 expression [63,64]. On the contrary, it was discovered that more IgA antibodies binding to HIV-1 correlated with an increased risk of infection [65,66]. Overall, this clinical trial demonstrates the importance of utilizing a proper viral vectored vaccine that is capable of inducing a combination of B cell, CD4+, and CD8+ T cell responses [64].

Another exciting development for poxvirus vectors is the development and application of recombinant FWPV vectors that have shown promising clinical and preclinical results, especially when they are used as part of a prime-boost vaccination strategy to achieve protective immunity. This is demonstrated through an HIV-1 vaccination trial, in which DNA expressing mutated HIV-1’s gag, pol, env, vpu, tat, and rev was first administered to human volunteers [65]. The HIV-1 genes were mutated to disable these HIV-1 proteins’ functions [66]. Volunteers received 1 mg of the DNA construct via intramuscular injection at zero- and four-weeks post-administration. Then, at eight-weeks post-administration, the volunteers received an intramuscular booster of the recombinant FWPV vector expressing the HIV-1 gag/pol genes at 5 × 10^7^ plaque forming units (pfu) [65]. The vaccine given in a prime-boost vaccination method was found to be safe, but there was no observable difference in gag or pol-specific T-cell responses between vaccinated and placebo recipients [65] despite eliciting both CD8+ and CD4+ T cell responses in non-human primate models [67,68]. In contrast, priming with recombinant canarypox virus [ALVAC-HIV (vCP1521)] and boosting with recombinant FWPV encoding the HIV-1 glycoprotein 120 (AIDSVAX B/E) resulted in partial control of HIV replication in some volunteers [64]. The differing results of these two clinical trials demonstrate the importance of utilizing FWPV viral vectors in a prime-boost regime as compared to other immunogenic vaccination methods. In studies of the immune response to FWPV viral vectors, it has been demonstrated that these vectors can stimulate a robust immune response in mice through engaging both the adaptive and innate immune arms, specifically the toll-like receptors TLR-7 and TLR-9 and the MyD88 (myeloid differentiation primary response gene 88) adaptor protein [69]. Engagement of these cellular receptors likely stimulates plasmacytoid dendritic cells (pDCs) to produce IL-18 cytokine, which drives T cell responses, while also eliciting a type I IFN response [69].

### 3.4. Alphavirus Vectors

**Figure 1:**
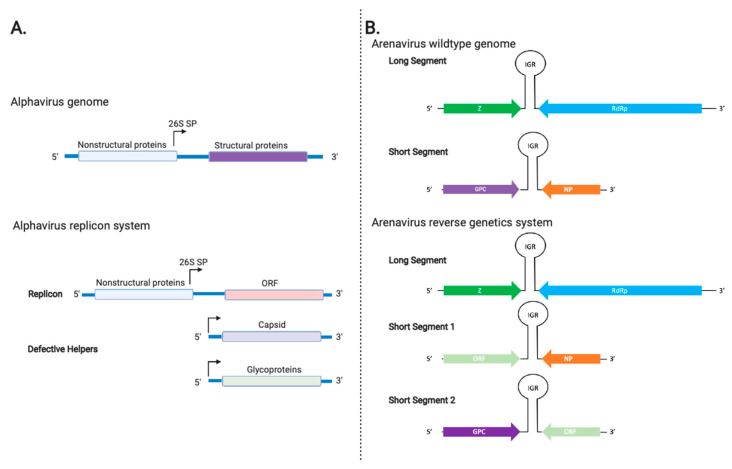
Schematic of alphavirus and arenavirus genomes and the genetic techniques to generate viral vectors from them. (**A**) Wildtype alphavirus genome (top) and its replicon system (bottom). The 26S is the viral subgenomic promoter. ORF: open reading frame (**B**) The wildtype bi-segmented genome of arenaviruses (top) and the reverse genetics system to generate the arenaviral tri-segmented genome (bottom). The ambisense genome contains the glycoprotein precursor complex (GPC), the nucleoprotein (NP), the matrix (Z), and the RNA-dependent RNA polymerase (RdRp). A noncoding intergenic region (IGR) separates the two genes, which are transcribed in opposite directions. Created with Biorender.

Sindbis virus (SIN), Semliki Forest virus (SFV), and Venezuelan equine encephalitis (VEE) virus from the family *Togaviridae* have also been developed as viral vectors. Alphaviruses are positive-stranded RNA viruses that have two open reading frames, one of which is accessible by a subgenomic promoter that encodes late viral genes (Figure 1A). Several features make these viruses attractive as viral vaccine vectors, which include but are not necessarily limited to these viruses having a low seroprevalence rate in the general human population, possessing an intrinsic ability to infect a broad range of animals [70], encoding their own structural proteins so they do not need a helper virus for viral packaging, and producing large amounts of the heterologous protein while growing to high viral titers [71].

As wildtype SIN and SFV are able to cause disease in humans, alphavirus vectors are most commonly designed as replicons. Replicons are single-cycle virus-like-particles (VLPs) that express the alphavirus proteins to assemble and bud off cells as virus-like structures [19]. Replicons for SFV [20], SIN [72], and VEE [73] have been similarly developed and designed. The replicons can express components of structural capsid proteins from these different viruses. Generally, the gene of interest (ORF) is placed downstream of the nonstructural (nsP1-4) genes in place of the structural gene and is transcribed by the 26S subgenomic promoter [19] (Figure 1A).

In order to minimize the potential of generating RC viruses in these virus preparations, two helper systems that have the genes encoding for the viral capsid and glycoprotein on two separate defective helper (DH) RNAs were developed [74,75,76]. To create the VLPs, the cells are transfected with both helper RNAs and the replicon. This approach has effectively reduced the generation of RC viruses below detectable levels [74,75,76]. After administration of the replicon-based vaccines, these VLPs particles infect cells that undergo apoptosis, and these apoptotic bodies with the vaccine antigen of interest are processed and presented to the CD4+ and CD8+ T cells by the antigen-presenting cells (APCs) [77]. VEE VLPs are lymphotropic [78] so that they can preferentially target dendritic cells for MHC class I presentation of the vaccine antigen to T cells to generate antigen-specific cellular immune responses [78]. In contrast, SFV and SIN are not lymphotropic, and as such, they induce cytotoxic T lymphocyte responses through cross priming, an important mechanism of APCs to present antigens on MHC class I to CD8+ T cells [79,80].

SIN, SFV, and VEE have all been used as vaccine vectors for a variety of diseases due to their large packaging capacity and promising immunogenicity (Table 1). For example, SFV has been used to develop vaccine candidates for HIV-1 [81], Murray Valley encephalitis virus [82], and influenza [83], and all these candidate vaccines have been tested in the appropriate mouse models. SIN has been used as a vaccine vector to develop vaccine candidates for *Plasmodium yoelli* for evaluation in mice [84], Hantavirus Seoul virus for testing in Syrian hamsters [85], and HPV for testing in mice [86]. VEE has been used as a vaccine vector to develop candidate vaccines for hemorrhagic fever viruses Lassa [19], Ebola [87,88,89], and Marburg viruses that have been tested in mice, guinea pigs, and/or macaques [90], as well as for simian immunodeficiency virus (SIV) for testing in macaques [91], and for Botulinum neurotoxin for testing in mice [92]. In general, these viral replicon-based vaccines induced durable and neutralizing antibody responses, lasting cytotoxic T-cell memory, and protection upon challenge [93]. Recombinant SFV has been shown to aid in the maturation and activation of skin Langerhans cells by increasing MHC11, CD54, and CD80 activating markers on these cells [94]. This cellular activation can contribute to the strong adaptive immune responses seen in preclinical trials. However, only recently have alphavirus vectors been used as viral vectors for vaccine development for some human diseases that have been tested in human clinical trials (Table 2 above). Alphavirus vectors have been shown to work well in prime-boost regimes but when administered on their own [95], there has been difficulty in replicating the robust immune responses seen in preclinical trials in humans [96,97,98], which calls for more attention to dose optimization and/or improved delivery methods [95].

### 3.5. Arenavirus Vectors

Two viral vectors derived from the virus family *Arenaviridae* (tri-segmented lymphocytic choriomeningitis virus (LCMV) and tri-segmented Pichinde virus (rP18tri)) have been developed through a reverse genetics approach, which will be discussed in some detail below (Table 1). These and other mammalian arenaviruses (mammarenaviruses) exist in nature as enveloped bi-segmented ambi-sense RNA viruses with four essential viral gene products encoded on their genome (Figure 1B above): the glycoprotein precursor complex GPC, the nucleoprotein NP, the matrix Z, and the RNA-dependent RNA L polymerase [99]. These arenaviruses are generally associated with rodent-transmitted zoonotic diseases that can result in viral encephalitis (e.g., LCMV) [100] or hemorrhagic fevers (e.g., Lassa virus as well as LCMV in some known cases of solid organ transplantation) [101]. However, it is noteworthy that the incidence of LCMV infection and disease pathogenesis in humans is real, albeit relatively low [102]. On the contrary, Pichinde virus (PICV) is not known to cause any human diseases [103] and is therefore thought to be safer for use as a viral vaccine vector [104].

As previously mentioned, all mammarenaviruses carry two RNA genomic segments in their virion particles. LCMV was the first arenavirus vaccine vector developed through reverse genetics technology [21]. The LCMV tri-segmented viral vector was generated through the separation of the GPC and NP genes, which are typically encoded on the short (S) genomic segment, and duplication of their associated regulatory RNA elements into two separate S segments (S1 and S2), each containing the GPC or NP gene and additional open reading frames (ORF) of the vaccine antigen(s) (Figure 1B). This ensures that there is a strong selective pressure to create a recombinant virus that contains all of the viral genes (Z, L, GPC, and NP) along with heterologous gene(s) as vaccine antigen(s). The tri-segmented LCMV genome has been shown to successfully express the reporter genes, i.e., the enhanced green fluorescent protein (eGFP) and the chloramphenicol acetyltransferase (CAT) gene [21]. This recombinant tri-segmented LCMV was found to exhibit reduced viral growth and attenuation in a mouse model, and upon a single intraperitoneal injection, it was able to completely protect immunocompetent mice against a lethal challenge with the wildtype LCMV [105]. Further testing remains to be done for the tri-segmented LCMV vector. Another approach to vaccine design utilizing the LCMV vector is the creation of RD LCMV via using a four-plasmid co-transfection system. In this system, the S and L RNA genomic segments are cloned into plasmids that are under control of the RNA polymerase I promoter and terminator to regulate the expression of most of the viral genes (Z, NP, L), except for GPC on the S segment, which is replaced by the heterologous gene. To optimize viral production, the viral NP and L are cloned into two separate plasmids for expression under the control of the RNA polymerase II promoter [106]. These four plasmids are transfected into cells that stably express the GPC gene product to generate recombinant RD rLCMV that can be amplified only in these cells, yet they contain all four viral gene products as well as any heterologous genes cloned into the viral genomic segment to infect a target cell for the purpose of expressing the heterologous gene (e.g., ovalbumin or OVA) [106]. Using this as a basis for vaccine design, a similar RD rLCMV was created by replacing the gene encoding for the LCMV GPC with SIVmac239 Env and Gag antigens for testing in mice and cynomolgus macaques, which showed induction of polyfunctional SIV-specific T cell and antibody responses against both dominant and subdominant antigenic epitopes [107].

In addition to the aforementioned LCMV vaccine vectors, a tri-segmented PICV (rP18tri) has been developed using a similar reverse genetics approach to encode the influenza nucleoprotein (NP) and hemagglutinin (HA) as modeled antigens [23,108]. The safety and immunogenicity of this rP18tri-based vaccine has been evaluated in mice. It has been shown to be attenuated in cell culture and mice and can induce strong levels of humoral and cell mediated immunity against these modeled antigens, which increase upon a booster dose in vaccinated mice, which is unique for a viral vectored vaccine [108]. Other advantages of the rP18tri vaccine vector include the extremely low seroprevalence (i.e., low preexisting immunity) of PICV in the general human population and, as it is not known to cause diseases in humans, this viral vector has a better biosafety profile than the LCMV vector. Additionally, PICV does not inhibit humans’ innate immune activator proteins (i.e., retinoic-acid inducible gene-I or RIG-I and melanoma differentiation-associated protein 5 or MDA5) as LCMV does, which aids its ability to mediate strong innate and adaptive immune responses against the immunogens [108]. These arenaviral vectors exhibit a naturally high tropism for APCs, which elicit a robust adaptive immune response while inducing very low levels of anti-PICV vector immunity. This is due in part to the relatively heavy glycosylation of the arenaviral GPCs (especially that of PICV), which can impair the development of specific antibodies to neutralize the viral vectors, making these arenavirus vaccine vectors ideal for use in a prime and boost vaccination strategy [109]. However, due to their status as a relatively new virus vector, these arenaviral vaccine vectors have not entered into any clinical trials. However, they hold great promise for clinical applications for vaccine development for use in veterinary and human medicines.

### 3.6. Herpesvirus Vectors

There have been a number of approaches taken to design virus vectors from the virus family *Herpesviridae*, which consists of large DNA viruses that can establish long, latent, and often asymptomatic infections [110]. One of the most commonly used herpesvirus vectors is cytomegalovirus (CMV). These CMV-based vectors have been developed mainly as live attenuated or RD vectors. CMV can induce a pronounced and long-lived CD8+ T cell response, which persists even in elderly human populations [111]. In addition to inducing immune memory formation, CMV vectors are able to superinfect or re-infect a previously exposed host, which is important due to the ubiquity of CMV, and, like poxviruses, they are able to accommodate a large amount of foreign genetic information (Table 1) [112]. While RC CMV vectors are able to elicit strong immune responses similar to adenovirus vectors, there are potential safety issues as maternal–fetal congenital transmission of human CMV (HCMV) can cause serious and sometimes fatal disease in children [27]. For these reasons, RD and spread-deficient herpesvirus vectors are favored as vaccine vectors that are able to induce a strong adaptive immune response without any potential adverse health risks to the vaccinated individual and/or her offspring.

Another approach for CMV vector development has been through attenuation of the virus by deleting viral genes that interfere with the host immune response or by inserting genes that increase its immunogenicity, thus maintaining the replication competence of the virus while improving its biosafety profile. As an example of this approach, Slavuljica and colleagues inserted the activating NK group 2, member D receptor (NKG2D) ligand retinoic acid early-inducible protein-1 γ (RAE-1γ) in place of the viral m152/gp40, which typically downmodulates NKG2D ligands on the surfaces of infected cells, on the mouse CMV (MCMV) viral genome [24]. This modification attenuated the virus in newborn and immunocompromised mice while still eliciting a strong and protective CD8^+^ T cell response to wildtype mice against MCMV [24]. Building on this work, another MCMV vector has been developed that expresses the NKG2D ligand murine UL16-binding protein-like transcript-1 (MULT-1) in place of the viral m145 gene, which has a similar function as the viral m152. This viral vector demonstrated a further level of enhanced safety profile in newborn mice than the MCMV viral vector containing the NK cell receptor NKG2D ligand RAE-1γ, while still maintaining a potent immune response against the virus [25].

Several CMV viral vectors have been tested for their efficacy as viral vaccine vectors for a variety of viral and bacterial diseases. The rhesus monkey CMV (RhCMV) vector expressing several SIV proteins has been found to induce a specific CD4^+^ and CD8^+^ response in a number of studies [113,114], resulting in clearance of a highly pathogenic SIV infection in macaques [114,115]. Two such vaccine candidates have been developed and tested for Ebola [116,117]. Additionally, a CMV vector has been proposed for use to develop vaccines for tetanus as the persistent immune response stimulated by this viral vector may overcome the need for the multiple booster shots necessary with the current vaccine [118]. Lastly, there have been two CMV vectored vaccines designed for tuberculosis that are based on MCMV [119] and RhCMV [120], with both viral vectors displaying protective immune responses in mice and rhesus macaques, respectively.

### 3.7. Flavivirus Vectors

Viral vectors derived from the virus family *Flaviviridae* include the live attenuated yellow fever virus 17D (YF-17D and YF-17DD strains), which this article will mainly focus on. Flaviviruses are spherical, enveloped viruses with a single-stranded RNA genome. Attempts to apply flaviviruses as viral vectors have largely focused on the modification of the YF-17D vaccine, which is considered one of the safest and most efficacious live attenuated vaccines [121,122]. This vaccine can produce neutralizing antibodies against yellow fever virus in recipients that last for at least 39 years [121,122]. YF-17D has been developed into two sub-strain vaccines (17DD and 17D-204) for manufacturing [28]. Analyses of the immune response by these two sub-strains, which share over 99% of their nucleotide sequences, show that they can stimulate similar immunogenicity [28]. In addition to its ability to induce long-lasting antibodies, YF-17D has recently attracted interest for its ability to elicit a polyfunctional and long-lasting CD8^+^ T cell response [29,123]. For these reasons, YF-17D has been developed as a viral vector to express heterologous gene(s).

There have been a variety of approaches taken to develop YF-17D into a viral vector platform to deliver heterologous gene(s). The first and most common approach is through the creation of a chimeric virus with other flaviviruses. The creation of a chimeric virus is possible due to the conserved features of flavivirus genome organization and replication [124]. To create a chimeric virus, plasmids were designed in which the structural proteins of the Japanese encephalitis virus (pre-membrane (prM) and envelope (E)) were inserted in place of the homologous yellow fever virus proteins within the YF-17D genome. The recombinant chimeric virus was plaque-purified and tested for safety in mice [125]. Similar approaches were developed for West Nile virus (WNV) [126] and dengue virus (DENV) [127]. These efforts have yielded a number of vaccines that are in clinical testing or have been FDA-approved for use in humans. For example, Sanofi-Pasteur developed Dengvaxia^®^, a live attenuated tetravalent vaccine which contains chimeras of prM and E genes of the four DENV types in place of the homologous genes of YF-17D [127]. This vaccine is currently in phase III of clinical trials and displays high protective efficacy against DENV1, DENV3, and DENV4 but poor protection against DENV2 [128,129,130,131,132]. Additionally, the ChimeriVax-JE vaccine is available for human use as it is safe and provides long-lasting immunity after a single vaccination dose against infection by Japanese encephalitis virus [133,134,135]. A WNV vaccine, ChimeriVax-WNO2, has been developed with specific mutations introduced in order to reduce neurovirulence and is in phase II of clinical trials [136]. Recently, a YF-17D-based vaccine for Zika virus, ChimeriVax-Zika, has been developed and is in preclinical testing in mice [137].

In addition to applications on closely related virus species, multiple attempts have been made to introduce heterologous genes from other pathogens into intragenic and intergenic sites on the YF-17D genome in order to generate other vaccine candidates against different diseases. The most successful insertion site is the envelope protein/non-structural protein 1 (E/NS1) intergenic region, where there is a shift from encoding the viral structural to non-structural genes. The insertion of a heterologous gene into this site is thought to result in fewer disturbances to the YF viral life cycle [138]. To do this, the first nine amino acids of the YF NS1 are duplicated and linked to the amino terminus of the heterologous protein. Then, the carboxyl terminus of the heterologous protein is fused to the YF-17D E protein stem anchor domain or other homologous proteins [138,139]. By doing so, protein antigens from SIV [140], *Trypanosoma cruzi* [141], HIV-1 [142], and Lassa fever virus [143] have been expressed from this YF genomic region and have shown strong T cell responses against these heterologous antigens following inoculation into multiple preclinical animal models, such as guinea pigs, mice, and macaques, depending on the pathogen that is targeted for vaccination. Attempts have also been made to insert heterologous gene(s) into the YF17-D C gene to create bi-cistronic viral RNA molecules [144,145], but the vaccine was found to be insufficiently stable. These approaches have been extensively reviewed elsewhere [146].

### 3.8. Paramyxovirus Vectors

There are two main groups of viruses in the *Paramyxoviridae* family that have been used for viral vector developments: those in the order *Mononegavirales* and those in the genus *Avulavirus*. Viruses from *Paramyxoviridae* are diverse and contain important pathogens, such as Rubeola virus and mumps viruses, which cause measles and mumps, respectively. These viruses are amenable for viral vector development because their genomes can be easily cloned and do not recombine, and they thus have a stable genome. Additionally, they have a broad host range.

There are two main viral vectors from the genus *Avulavirus*: avian paramyxovirus serotype-1 (APMV-1) and APMV-3 (Table 1). APMV-1 contains all strains of Newcastle disease virus (NDV), which can cause enormous losses in the poultry industry [147]. For this reason, viral vectors based upon NDV need to be attenuated for poultry while being immunogenic for human use. Through the use of the reverse genetics technique, NDV has been engineered into a safe and immunogenic viral vector that has been used for both human and veterinary purposes [148]. Briefly, cDNA constructs of the virus were designed to include restriction enzyme cut sites to create and accommodate an open-reading frame (ORF) of the heterologous gene between the region encoding the viral phosphoprotein (P) and matrix (M) protein. These viral constructs have been utilized to design vaccines for a variety of diseases and tested preclinically, including Rift Valley fever virus [149] and bovine herpesvirus-1 in calves [150], EBOV in rhesus macaques [30], and infectious laryngotracheitis virus [31] and H5N1 avian influenza virus [151] in chickens.

APMV-3 has been proposed as a more attractive candidate for its ability to infect non-human primates without causing clinical disease, which suggests a favorable safety profile for human use [152] and its ability to replicate better than other APMV serotypes in a wide range of cell types. Additionally, unlike APMV-1, APMV-3 is not a select agent [152], which makes it easier and safer to use. Similar to APMV-1, a reverse genetics approach has been used to create an ORF of the heterologous gene between the regions encoding the viral P and M genes [152]. Recently, a similar vaccine based on APMV-3 has been developed through the insertion of genes expressing the EBOV glycoprotein (GP) at various sites on the APMV genome [153]. It was found that the highest expression of the EBOV GP was from insertion of this heterologous gene between the APMV nucleoprotein (N) and P rather than between M and P, suggesting that the optimal location for the heterologous gene on the APMV genome may vary depending on the antigens. The vaccine has also been found to elicit humoral immune responses, specifically IgG and IgA, in guinea pigs [154]. However, in this study, the researchers used rNDV-3FHN, a modified NDV vector expressing ectodomains of APMV-3 glycoproteins, which is based on APMV-1, and this viral vector was found to be better at inducing neutralizing antibodies against EBOV in comparison to the rAPMV-3 vector [153]. These results indicate that further research on the design and application of paramyxovirus vectors is warranted.

### 3.9. VSV and Rabies Virus Vectors

VSV and rabies virus are members of the *Rhabdoviridae* virus family [155]. These enveloped viruses have a negative-strand RNA genome and have been developed into viral vectors. In general, the advantages of rhabdovirus vectors include their cytoplasmic replication strategy as well as their modular genome organization which is conducive for genetic manipulation [155] (Table 1).

VSV is known for its ability to induce robust antigen-specific neutralizing antibody responses as well as a modest T-cell-mediated immune response [156]. Additionally, whereas preexisting immunity in the general population is a concern for some of these viral vectors, VSV typically infects animals but has caused only rare outbreaks in humans so that there is a low likelihood of the general population having preexisting immunity to the VSV-based viral vector [156]. VSV has typically been developed as a minimally attenuated RC vector for vaccine development purposes, for which it has demonstrated safety and immunogenicity in multiple clinical trials [9]. There are some main approaches to genetically modify and attenuate VSV as a viral vector for vaccine development. The first is the replacement of the gene encoding the viral glycoprotein (G) of VSV with a different viral glycoprotein gene product to recognize a certain viral receptor on the target cells in order to alter its cellular tropism, a process known as pseudotyping. Another attenuation method that has shown success in vivo and in vitro is the truncation of the cytoplasmic tail of the G protein [9]. This attenuation method is thought to work by impairing the interaction of the cytoplasmic tail of G protein with underlying viral core proteins, thus impacting virus particle maturation and budding [157]. Another attenuation mechanism is mediated through genetic manipulation to downregulate the expression of one or more key viral structural proteins, such as the nucleocapsid (N) gene or the matrix (M) protein [32,33].

Using these principles, rVSV-ZEBOV, a recombinant, replication-competent VSV that expresses the glycoprotein of the Zaire EBOV, was created and evaluated for efficacy and safety in humans [158]. The vaccine was reported to have an efficacy of 100% during a ring-vaccination strategy [158], but this efficacy has recently been disputed [159,160]. Regardless, this rVSV-ZEBOV has been FDA-approved for human use [42], and it represents the first and the only viral vectored vaccine that has been approved for human use (to the best of our knowledge). Through in-depth studies of the immune responses generated by this rVSV-ZEBOV vaccine, researchers have determined important immune signatures that can be monitored in future VSV clinical trials or other viral vectored vaccine clinical trials. For example, by analyzing blood samples at various time points after receiving the rVSV-ZEBOV vaccine, it was found that increased plasma concentration of interferon-γ-inducible protein 10 (IP-100 and frequency of CD56^bright^ NK cells at day 3 post-vaccination positively correlated with a strong antibody response to the vaccine [161]. Conversely, an increased frequency of CD86+ myeloid dendritic cells and monocytes correlated with a dampened antibody response [161]. These immune correlates can be used to predict the success of potential vaccines and can also be used in future vaccine studies with a VSV vector in which a strong antibody response is desired. The success of VSV vectors has prompted further studies and it is currently being tested as a vaccine for a variety of other viruses, including Zika virus [162], HIV-1 [163], and others. These VSV-based vectors have been extensively reviewed elsewhere [164].

A unique aspect of rhabdoviruses, such as rabies viruses (RABV), is that they infect neurons via their axonal terminals. The virus then spreads exclusively in a retrograde fashion, spreading from the axon terminals of neurons to the cell body and to other synaptically connected neurons. Therefore, it is convenient to use this viral vector to understand neuronal trafficking [165,166] across synapses if its glycoprotein (G) remains intact or is provided in trans [167]. Similar to VSV, RABV can also be pseudotyped with non-native envelope glycoproteins, which can alter and broaden its cellular tropism [167,168]. However, a potential limitation of the RABV vectors is the size limitation of insertion of the heterologous gene. That being said, some RABV-based vectors have been shown to stably express up to 4.4 kB of insert, which is more than 50% of their genome size [168]. RABV vectors have been used for vaccine development against different pathogens, including HIV-1 and Nipah virus [169,170,171,172]. In the Nipah vaccine study, an RC RABV-based vaccine expressing the Nipah virus (NiV) glycoprotein (G) was created for evaluation in mice. The vaccine induced a strong neutralizing antibody response that was also cross-reactive against a related Hendra virus [172]. Specifically, the antibodies were mainly IgG2c, which suggested a Th1-biased response [172]. However, as this study was conducted in mice, a challenge study evaluating the protective efficacy against NiV utilizing this vaccine vector will need to be conducted in an animal model that is susceptible to NiV.

Due to general concerns of reversion to virulence with an RC RABV vector, an SC-RABV vector has been developed. This was accomplished through deleting the RABV G gene from the viral genome [34]. The viral G glycoprotein plays a critical role in mediating viral attachment and entry into permissive cells. Similar to the creation of the SC-Ad, the virus particles are produced by transfecting cells that express the viral G glycoprotein in trans so that virus particles can be made during cell culturing. When given as a vaccine, these virus particles can infect their target cells but are not able to produce infectious virus particles and instead just transcribe viral RNA; as such, without G, RABV infection is limited to a single cycle [34,35,173]. The SC-RABV vector has been shown to induce a robust cytotoxic T cell response in mice [34]. The promise of RABV vectors to induce a strong Th1 type immune response and new innovations in SC rabies vector development warrant further consideration.

## 4. Viral Vaccine Vectors in Veterinary Medicine: A Story of Success

Viral vaccine vectors have been tested and approved for a number of veterinary uses [174,175]. For a complete list of USDA-approved products, please follow this web-link: https://www.aphis.usda.gov/animal_health/vet_biologics/publications/currentprodcodebook.pdf. In contrast, only a single viral vector, the aforementioned rVSV-ZEBOV, has been approved for human use [42]. This discrepancy can be attributed to many factors that include but are not necessarily limited to the stringent and time-consuming licensing process for human vaccine development, which usually consists of costly and lengthy human clinical trials. A number of the previously discussed vectors have been investigated for use in developing vaccines for animals, including poxviruses, herpesviruses, adenoviruses, flaviviruses, and paramyxoviruses [175,176]. Of these, poxvirus and herpesvirus vectors are currently approved for use by the United States Department of Agriculture’s (USDA) Animal and Plant Health Inspection Service (APHIS) [177]. Approved ALVAC vectors in companion animals include vaccines for canine distemper virus (dogs and ferrets), feline leukemia virus, and RABV (cats) [177,178,179,180,181] (Table 3 above). As discussed previously, ALVAC vectors are advantageous in that they can accommodate large amounts of heterologous DNA as vaccine antigens, elicit strong humoral and cellular immune responses, and do not replicate in mammalian cells. As an example, the ALVAC-vectored recombinant RABV vaccine induces high antibody titers in cats for up to three years following a standard vaccination program [181]. Additionally, the ALVAC-vectored rabies and feline leukemia vaccines are protective in the absence of an adjuvant, which improves their safety profiles. This is especially important for cats, where adjuvant-based vaccines are a risk factor for feline injection site sarcomas [182]. Accordingly, the European Advisory Board on Cat Diseases recommends use of non-adjuvanted, live attenuated, or recombinant vaccines for cats [183].

Viral vectors have been particularly effective for wildlife rabies vaccination campaigns. Recombinant vaccinia virus expressing the RABV G glycoprotein is stable, effective, and safe when administered orally [5,6] (Table 3). Adult foxes developed high neutralizing antibody against rabies and resisted challenge at 28 days post-vaccination, with immunity lasting at least 18 months in adults and 12 months in cubs [5]. Efficacy has also been established for a number of other target species, including coyotes, raccoons, and skunks [184,185]. No adverse side effects have been shown in target species or non-target species, such as rodents, companion animals, raptors, and non-human primates (as a model for human exposure) [184] that may be exposed accidentally. In comparison, inactivated and live attenuated vaccines may be ineffective and, in the case of attenuated vaccines, have the potential to revert to virulence. This highly successful vaccine strategy has dramatically reduced or eliminated sylvatic rabies in portions of North America and Europe [184], which has important public health implications.

In addition to companion animals and wildlife, viral vectors are also widely used in production animal settings. Simple and cost-effective vaccination strategies are especially important in the poultry industry, where a number of infectious diseases may lead to massive production losses. For example, during the 2014–2015 outbreak of highly pathogenic avian influenza virus, an estimated 48 million chickens and turkeys in the United States died of the disease or were destroyed, resulting in estimated economic losses of USD 3.3 billion [186]. Turkey herpesvirus (HVT), which is non-pathogenic in chickens, has been used for decades as a safe and effective vaccine for Marek’s disease [187]. As previously discussed, herpesviruses can accommodate large segments of heterologous genes, so recombinant HVTs have been engineered to express antigens from a variety of infectious agents. For example, chicks inoculated with HVT expressing the fusion protein from NDV were protected against lethal velogenic NDV and Marek’s disease virus challenge [188]. For this vaccine, the fusion protein was inserted into the unique short element of the HVT genome and regulated by a promoter element derived from the Rous sarcoma virus long terminal repeat. Another recombinant HVT vector vaccine carrying the US6 and US7 genes of infectious laryngotracheitis virus (ILTV) induces an antibody response against glycoprotein I of ILTV in vaccinated chickens and reduces clinical signs following challenge [189]. HVT vectors have also been used to vaccinate against infectious bursal disease virus (IBDV), which is a highly contagious and economically important disease in the poultry industry. Vaccines have been developed that express the VP2 gene of IBDV at the glycoprotein L gene locus of HVT, which provides protection against a lethal challenge [190] (Table 3). This vaccine elicits high neutralizing serum antibodies and does not cause bursal lesions, in contrast to some modified live vaccines [191]. In experimental [191] and field efficacy trials [192], the HVT-IBD vaccine protects against challenge with very virulent IBDV in the presence of maternal antibodies. While the HVT-IBD vaccine is very effective in controlling IBD, there are limitations when using multiple HVT vectored vaccines in the same animal due to immune interference. Additional vectors are therefore needed in order to expand the avian vaccine portfolio. Gallid herpesvirus-3 strain SB-1, commonly used as a live attenuated vaccine to control Marek’s disease, has recently been developed as a vector expressing VP2 of IBDV [193]. Chickens vaccinated with the recombinant SB-1 had high neutralizing antibodies comparable to the commercial HVT-IBD vaccine and were protected against lethal challenge with very virulent IBDV. Lastly, recent advances in CRISPR/Cas9 gene editing have been applied to generate HVT vectors expressing VP2 of IBDV [194], as well as a triple insert carrying VP2, glycoprotein D-glycoprotein I of ILTV, and H9HA of avian influenza virus [195]. Gene editing techniques are an exciting addition to vaccinology, with the potential to rapidly generate effective, multivalent poultry vaccines.

Despite these advantages, vectored vaccines may be more labor intensive and costly to manufacture when compared to conventional non-viral vectored vaccines [9,196], which may potentially limit their use in production animal settings. Additionally, like their live attenuated and inactivated counterparts, viral vectored vaccines can be inhibited by the presence of maternal antibodies against the vector itself or the target antigen. As an example, raccoon kits born to mothers vaccinated with the oral vaccinia-vectored rabies vaccine had maternally-derived antibodies that appeared to interfere with the efficacy of subsequent oral vaccination [197]. Kits without maternally derived antibodies, however, mounted an appropriate immune response upon vaccination. Canine distemper virus (CDV), a highly fatal disease in dogs, ferrets, and wildlife, is well-controlled by live attenuated vaccines. Vaccine failure due to passive immunity in young puppies is common, so viral vector-based strategies have been developed. Adenovirus vectors have been demonstrated to be effective in the presence of maternal antibodies against the vector [198,199], prompting the investigation of replication competent canine adenovirus-2 (CAV2) for possible use in CDV vaccination. Puppies born to dams with immunity to CAV2 mounted a protective immune response when vaccinated subcutaneously with a CAV2-vectored CDV vaccine but failed to overcome preexisting vector immunity when vaccinated intranasally [200]. The authors of this study hypothesized that an active CAV2 infection with a strong local immune response interfered with mucosal vaccination. An alternative ALVAC vector carrying the HA and F proteins of CDV has also been developed that provides protection from disease in the presence of maternal antibodies, as there is no preexisting immunity to the vector [178]. Therefore, this ALVAC-vectored vaccine is widely used for protection against CDV.

Vaccine development for veterinary populations confers the additional advantage of using animals as human vaccine models, which is especially relevant for large animal models. Bovine tuberculosis has features similar to the human disease, so cattle are considered a natural model for disease pathogenesis and vaccine development [201]. Cattle primed with *Mycobacterium bovis* bacillus Calmette–Guerin (BCG) vaccine followed by boosting with either an MVA or RD adenovirus vector expressing the mycobacterial antigen Ag85A had significantly reduced lung pathology, bacterial loads, and increased memory immune responses compared to BCG alone [202]. Similar prime-boost vaccination strategies using RD adenoviruses and MVA vectors are currently undergoing human clinical trials [203], so cattle may serve as a useful preclinical model of naturally occurring tuberculosis. Dogs are also a physiologically relevant large animal model of naturally occurring genetic diseases which are a potential target for viral vector-based gene therapies. For example, in a preclinical model of hemophilia A, dogs had sustained expression of clotting factor VIII delivered via an adeno-associated virus vector [204].

Viral vectors play an important role in veterinary and public health. These vaccines provide effective and safe protection against companion animal diseases, zoonotic diseases, and livestock diseases, with significant economic impacts. Vector vaccines are particularly useful as differentiating infected from vaccinated animal (DIVA) vaccines, especially for the eradication of reportable livestock diseases. Because viral vectored vaccines can stimulate neutralizing antibodies to a limited number of expressed antigens, the immune response can be differentiated from that of a natural infection. One example of this is a RD-Ad5 vectored foot and mouth disease vaccine that has a conditional license from the USDA for emergency use [205]. Additionally, large animals are increasingly recognized as biologically important preclinical models of vector-based therapies for experimental and natural disease. Veterinary medicine is at the forefront of viral vectored vaccinology, so continued efforts to bring vector-based vaccines to the veterinary market are critical to advancing the technology and improving both human and animal health.

## 5. Examples of Application of Viral Vectored Vaccines for Zoonotic Infection of a High-Consequence Pathogen, SARS-CoV-2, the Causative Agent of COVID-19

Viral vectored vaccine development is crucial to respond to rapidly emerging zoonotic pathogens in order to produce to a diverse pool of vaccine candidates for immediate testing, especially considering that vaccine candidates have a 33% success rate of progressing from phase I of clinical trials to approval [206]. The urgency of vaccine development to curb the transmission of SARS-CoV-2, the causative agent of COVID-19, has produced a number of viral vaccine candidates that are currently in clinical testing. The most prominent examples are several adenovirus-based SARS-CoV-2 vaccine candidates. The RD chimpanzee adenoviral vaccine AZD1222 (formerly known as ChAdOx1 nCov) from Oxford University entered phase III clinical trials in August 2020 (Table 4) [207,208].

The same adenoviral backbone was previously used by the same group to produce a vaccine against Middle East respiratory syndrome coronavirus (MERS-CoV), which causes Middle East respiratory syndrome, by encoding the MERS-CoV spike (S) protein. Because this vaccine protected against six strains of MERS-CoV in rhesus macaques [209], this vaccine design is thought to have the potential for developing a vaccine for SARS-CoV-2. AZ1222 induced humoral and T cell-mediated immune responses in phase I/II clinical trials and did not induce any severe side effects [207]. AZD1222 also induced a humoral, CD8, and Th1-dominant CD4 response in mice and rhesus macaques. Notably, both a prime and a prime-boost dosing schedule prevented the development of pneumonia in rhesus macaques. However, unvaccinated and vaccinated macaques that were challenged with SARS-CoV-2 had no difference in the amount of nasal viral shedding [208]. It should be noted that the phase III clinical trial of the AZD1222 was paused in September 2020 after a participant in the UK trial developed a severe and unexplained illness [210]. The trials have since resumed in the UK but not yet in the US for undisclosed reasons.

Several additional RD adenoviral vectored SARS-CoV2 vaccines have also started clinical trials. Currently in phase I/II clinical trials, the Ad5-nCoV vaccine from CanSino Biologics induced robust humoral and T cell responses with a single dose of vaccination and showed only rare instances of moderate to severe side effects that were present mostly in the groups receiving a higher dose [211]. Following the release of these data, the Chinese government approved this vaccine for use in its military members. The Ad26.COV2.S vaccine candidate from Johnson and Johnson also resulted in robust antibody and T cell responses in rhesus macaques with a single dose of immunization, and upon viral challenge, lower viral titers were observed in animals with higher antibody titers [212]. The phase III trial of the Ad.26.COV2.S vaccine was also paused in October 2020 due to an unexplained serious illness [213]. The Gam-COVID-Vac adenoviral candidate from the Gamaleya Research Institute of Epidemiology and Microbiology was the first SARS-CoV-2 vaccine to gain approval from the Russian government for use in the general public before phase III clinical trials had begun [214,215]. Finally, the MVA gorilla adenovirus-vectored candidate from ReiThera began phase I clinical trials in Italy in August 2020 [216] and the VXA-CoV2-1 adenovirus type 5 vaccine candidate from Vaxart began phase I clinical trials in September 2020 [217].

Several vaccines based on other viral backbones have also started clinical trials. The TMV-083 live attenuated measles candidate from the Institut Pasteur expresses a modified SARS-CoV-2 surface glycoprotein (spike) and entered phase I clinical trials in August 2020 [218]. Additionally, the V591 and V590 live attenuated measles candidates from Merck also entered phase I clinical trials in August and September 2020, respectively [219,220]. Finally, the MVA-SARS-2-S Vaccinia Ankara vectored candidate from the Universitätsklinikum Hamburg-Eppendorf entered phase I clinical trials in Germany in September 2020 [221]. In addition to these viral vectored COVID-19 vaccine candidates that are in various stages of clinical trials, many other COVID-19 vaccine candidates, including non-viral vaccines, are also currently in preclinical development and clinical testing and have been reviewed elsewhere [196,222].

## 6. Summary

Many different viral vectors have been investigated and will remain to be important avenues in advancing the development of vaccines against many human and animal diseases, including those with zoonotic potential to cause global pandemics, yet each with their own advantages and disadvantages. For example, DNA viruses such as poxviruses and herpesviruses are able to accommodate large heterologous inserts of vaccine antigens as compared to the relatively small capacity for doing so with some RNA viruses. However, RNA virus replication, other than that of retroviruses and lentiviruses, is restricted to the cytosol, so the risk of host genomic integration is low. Lentiviruses and rhabdoviruses confer the additional advantage of pseudotyping, whereas heterologous viral envelope glycoproteins can be incorporated into the vector in order to broaden and/or alter cellular tropisms. A number of viral vectors have been developed that have low seroprevalence in the human population (e.g., SIN, SFV, VEE, VSV) or are non-pathogenic in humans (e.g., PICV and avipoxviruses), which improves their safety profiles. Choosing the most appropriate viral vector for vaccine development depends on the target species as well as the characteristics of the viral vector and/or pathogen. Major advantages of viral vectored vaccines are that they are capable of eliciting robust immune responses that are not always produced through other vaccine approaches and that they make adjuvant use obsolete. The need to consider new technologies for vaccine development is illustrated by public health threats, such as HIV-1, tuberculosis, and malaria, which, despite intensive research for decades, continue to be major public health burdens that elude effective preventative measures.

## Figures and Tables

**Table 1:** Known viral vectors used in vaccine development.

Virus Type	Retrovirus and Lentivirus	Adenovirus	Poxvirus	Alphavirus	Arenavirus	Herpesvirus	Flavivirus	Paramyxovirus	Rhabdovirus
Forms in development	Replication-defective Integrase-defective Single-cycle	Replication-competent Replication-defective	Replication-defective Replication-competent	Replication-competent Replication-defective Single-cycleReplicon	Reverse genetics system Replication-competent	Replication-defective Single-cycle	Replication-competent	Reverse genetics system Replication-competent	Replication-competent Single-cycle
Commonly used vectors	Moloney murine leukemia virus vector	RD-Ad5 SC-Ad6	Modified vaccinia virus Ankara Fowlpox Canarypox	Sindbis virus (SIN) Semliki Forest virus (SFV) Venezuelan equine encephalitis (VEE)	Lymphocytic choriomeningitis virus Pichinde virus	Cytomegalovirus Turkey herpesvirus	YF-17D Yellow fever virus 17D (YF-17D)	Avian paramyxovirus serotype (APMV)-1 APMV-3	Vesicular stomatitis virus (VSV) Rabies
Main advantages	Large packaging capacity Integrating ability Transducing non-dividing cells Ability to be pseudotyped	Broad tropism Variety of systemsdeveloped and tested Strong gene expression	Large packaging capacity Ability to induce a strong cellular immune response Broad host range	Broad tropism Ability to produce a large amount of heterologous protein while maintaining high titers	Low seroprevalence Ability to induce low antiviral immunity Targeting and infection of antigen presenting cells	Large packaging ability Capable of superinfecting a host Induces a long-lived T cell response Ease of manipulation	Ability to induce strong and long lasting adaptive immune response Relatively broad tropism	Does not undergo recombination so the vector is genetically stable Broad tropism Replication is generally limited to the respiratory tract	Ability to induce a robust humoral immune response Lack of preexisting immunity in generalpopulation Ability to be pseudotyped
Disadvantages	Concerns over insertional mutagenesis	Preexisting immunity to human adenoviral species like Ad5	Inability to induce strong immune responses in clinical trials	Transient gene expression so not useful for diseases that require long-term therapeutics	Needs further testing to ensure safety in humans	Causes lifelong infections in hosts so needs to be attenuated	Low immunogenicity of recombinant vectors and vector instability	Needs further testing to ensure safety in humans	Potential of neuro-virulence for rabies virus vector
Insert capacity	8 kB	8 kB	>25 kB	18 kB	4 kB	>30 kB	6 kB	4.5 kB	6 kB
References	[7,8,9]	[10,11,12,13,14]	[15,16,17,18]	[19,20]	[21,22,23]	[24,25,26,27]	[28,29]	[30,31]	[32,33,34,35]

**Table 2:** Human clinical trials in progress with viral vectored vaccines.

**Virus Vector**	**Phase I Clinical Trial**
**Poxviruses**	
MVA (Modified vaccinia virus Ankara)	Ebola, HIV, Hepatitis C, MERS-CoV
FPV (Fowlpox vector)	HIV
ALVAC (canarypox vector)	HIV
**Adenoviruses (Ad)**	
ChAd3 (Chimpanzee adenovirus)	Ebola Zaire, Hepatitis C, Ebola Sudan, Ebola Marburg
ChAdOx (Chimpanzee adenovirus)	Tuberculosis, Chikungunya, MERS-CoV
Ad5 (Adenovirus type 5)	Cystic fibrosis, HIV, Ebola Zaire
VXA (Replication-deficient Ad5)	Respiratory syncytial virus, Norovirus, Influenza
rAd26 (Recombinant Ad 26)	HIV, Ebola Zaire
Ad35	Tuberculosis, HIV
Ad4	HIV, Anthrax
**Vesicular Stomatitis Virus (VSV)**	
Replication-competent VSV	HIV
**Alphaviruses**	
VEE Replicon (Venezuelan equine encephalitis)	CMV
**Virus Vector**	**Phase II Clinical Trial**
**Poxviruses**	
MVA	CMV, Tuberculosis
**Adenoviruses**	
ChAdOx1	Malaria, SARS-CoV-2
ChAd63 (Chimpanzee adenovirus vector)	Malaria
VXA	Seasonal influenza
Ad5	Ebola, HIV, Pandemic influenza
Ad35	Malaria
**Virus Vector**	**FDA-Approved**
Replication-competent VSV	Ebola Zaire (ERVEBO^®^)

**Table 3:** Veterinary viral vectored vaccines licensed and available for commercial use in the United States.

Species	Pathogen/Disease	Antigen	Product	Manufacturer
**Canarypox Vector**				
**Dog**	Canine distemper virus	HA and F glycoproteins	RECOMBITEK	Boehringer-Ingelheim
**Cat**	Feline leukemia virus (FeLV) Rabies virus	Env, gag, pol Glycoprotein G	PUREVAX FeLV PUREVAX Rabies	Boehringer-Ingelheim
**Vaccinia Vector**				
**Raccoons, coyotes**	Rabies virus	Glycoprotein G	Raboral V-RG	Boehringer-Ingelheim
**Alphaherpesvirus (HVT) Vector**				
**Chicken**	Infectious bursal (IBD), Marek’s, Newcastle disease (ND) ND and Marek’s disease IBD and Marek’s disease Marek’s disease and infectious laryngotracheitis (LT)	VP2 of IBDV, F glycoprotein of NDV F glycoprotein VP2 of NDV Glycoprotein B	VAXXITEK HVT + IBD + ND Ultifend IBD ND NEWXXITEK HVT + ND VAXXITEK HVT + IBD Vectormune HVT IBD Vectormune LT	Boehringer-Ingelheim Ceva Boehringer-Ingelheim Ceva Ceva

**Table 4:** Viral vectored vaccines currently in development for SARS-CoV-2 *.

Vaccine Name	Vaccine Vector	Company and Country	Preliminary Results
AZD1222 (ChAdOx1 nCoV-19)	Adenovirus	Oxford University, UK	Phase I/II clinical trials showed that vaccine did not induce severe side effects and induced humoral and cell-mediated responses; Vaccine was found to induce humoral, CD8, and Th1-predominant CD4 responses in mice and rhesus macaques, and both a prime and prime-boost schedule protected rhesus macaques from development of pneumonia; Entered phase III clinical trials in August 2020; Trials were paused in September 2020 due to unexplained serious illness. Trials have resumed in the UK but not in the USA
Ad5-nCoV	Adenovirus	CanSino Biologics, China	Phase I/II clinical trials showed that vaccine induced antibody and cell-mediated responses with a single dose and did not induce severe side effects; Entered phase III clinical trials in August 2020; Approved by the Chinese government for its use in its military
Ad26.COV2.S	Adenovirus	Johnson and Johnson, USA	Induced antibody and T cell responses in rhesus macaques with a single dose and lower viral titers were found in animals with higher antibody titers; Entered phase III clinical trials in August 2020; Trials were paused in October 2020 due to unexplained serious illness
Gam-COVID-Vac	Adenovirus	Gamaleya Research Institute of Epidemiology and Microbiology, Russia	Approved for use in the general public by the Russian government before the release of clinical trial data and before phase III clinical trials had begun; Entered phase III clinical trials in August 2020;
GRAd-COV2	Adenovirus	ReiTherra, Italy	Entered phase I clinical trials in August 2020.
VXA-CoV2-1	Adenovirus	Vaxart, USA	Entered phase I clinical trials in September 2020;
TMV-083	Measles	Institut Pasteur, France	Entered phase I clinical trials in August 2020
V591 and V590	Measles	Merck, USA	Entered phase I clinical trials in August and September 2020
MVA-SARS-2-S	Vaccinia Ankara	Universitätsklinikum Hamburg-Eppendorf, Germany	Entered phase I clinical trials in September 2020

* COVID19 vaccine data compiled with the aid of the BioRender COVID19 Vaccine and Drug Tracker: https://biorender.com/covid-vaccine-tracker.
